# An empirical analysis of land property lawsuits and rainfalls

**DOI:** 10.1186/s40064-015-1659-2

**Published:** 2016-01-04

**Authors:** Li-Chen Chou, Chung-Yuan Fu

**Affiliations:** Department of Economics, City College of Wenzhou University, Chashan University Town, Wenzhou, Zhejiang China; Department of Economics, National Chengchi University, Taipei, Taiwan China

**Keywords:** Japanese colonial rule, Land property lawsuits, Rainfalls, 2SLS

## Abstract

This article using the database of Taiwanese land property lawsuits studies the economic effects of rainfalls on land property lawsuits during the period of *Japanese colonial rule* (1920–1941). The results obtained from basic ordinary least squares indicate that it shows no significant influences. However, an interesting result is that, when we adopt the approach of two stage least squares and use the variables of temperature and evaporation as the instrument variables of rainfalls, we find that there are highly significant influences on the lawsuits of land property. If 1 year comes with low average rainfalls, it means that the costs of productive inputs increase, because the available natural resource will decrease, and brings the distorted using of land property.

## Background

Among developing and developed countries, agricultural productivity is an important and decided factor of economic growth. Due to a higher ratio of first-order industries, hence, a stable growth rate of the agricultural productivity represents not only the economic growth of countries but also a raise in income per capita. Supporting the agricultural development depends on expanded areas of land and a coordination with climates. Because lands are a scarce factor in agricultural productive processes, however, given these explicit conditions of the size and the technology in these countries, an expanding of agricultural outputs is restrictive. Hence, the climate stability plays a key role on economic growth.

The economic outputs in agricultural societies highly depend on rainfalls. When the amount of rainfalls is not changing rapidly, the economic outputs will stably grow and then promote an increase in population. In most situations, the growth rate from the agricultural lands is always lower than that from the population, and in turn people would care about their own land property so that more and more disputes from land lawsuits occur in these countries. Therefore, if fluctuations of rainfalls maintain to be more stable, the number of land lawsuits may be decreased.

In the economic literature, there are many extended discussions and researches using rainfalls as an instrument variable or critical factor since (Miguel et al. 2004), which is study the relation between the national economic and the civil conflicts on Sahara area in Africa. The results from their pioneer paper pointed out that the civic conflicts do not be taken place by the residents that work within productive industries with higher returns. The reason is that the opportunity costs or the losses from the civic conflicts are too larger.

With the natural variables as sunshine durations, droughts, and rainfalls, some researches focused on the economic impacts in Africa (Ciccone 2011; Henderson et al. 2012; Hodler and Raschky 2014) and the impacts in nation democracy transformation (Brückner and Ciccone 2011). These researches figured out a fact that the rainfalls variable is a key factor on effects between economic activities, social stability, and degree of national democracy development. For example, Brückner and Ciccone (2011) investigated the political changes in these countries located on Sahara area and found that with the heavy volatility of rainfalls, the policymakers will partly open the political environment in order to the residents can attend and join the parties, and then the degree of democracy will be risen. André and Platteau (1998) studied the fact of Rwandan genocide and indicated that the events of civic wars or race fighting for resources will rise due to the rapidly increased times from extreme climate changes. The authors also pointed out that, in Rwandan, some causal relations between climate changes, legal or illegal land lawsuits, national wars and ethnic massacres are existed. Here, we wonder that rainfalls would effectively work on economic variables at these countries with higher average rainfalls in subtropical or tropical regions.

On the other side, from accompaniments of the development of economic market, trade between the products and the definition of property rights are tending meticulously. Buoye (2000) found out that many land lawsuit disputes and violence incidents actually are caused by a growing development of economic market, which pushes Chinese government pursuing clear land property ownership, when he surveys the China criminal database in 18 centuries.

According to the above arguments, are rainfalls also playing an influential reason in the areas, which are in the beginning of its modern economic development and just germinating concepts of related property? In other words, the disputes of land property are response to the frequency of land lawsuits, which is relative to rainfalls and scarcity of recourses.

This article studies the empirical influences on land property lawsuits in relations to rainfalls, according to the Taiwanese database during the period of Japanese colonial rule (1920–1941). The object of this article focuses on the Taiwanese data because the Taiwanese modern economic activities began in the period of Japanese colonial rule. The pattern of economic development in Taiwan is a transformation from the pure base of agricultural industries to the mixed stage of agricultural and light industries. Mainland China is also under the transformation of modern economy at the same time, but, unfortunately, it lacks a full database for statistics of the lawsuits and focuses on only some specific regions (Xiao et al. 2014). However, Taiwan is located on the subtropical region, and we hope that this article also applies to estimate other influences of rainfalls on different economic patterns in other subtropical regions.

## The data of the Land lawsuits, rainfalls and population

**Fig. 1:**
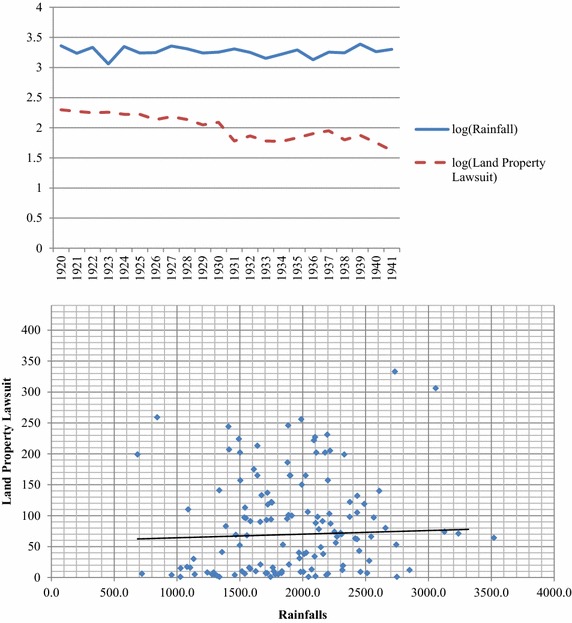
Rainfalls and land property lawsuit

The empirical data from Taiwanese database, built by National Taiwan University, is available for legal studies. We handy collected 128 samples from this database, and Table 1 reports descriptive statistics and correlations for these variables. These variables include the land property lawsuits (reflected the land property or the trade disputes), rainfalls over the empirical years (unit: millimeter), population, east region dummy (reflected the difference of economic development in different areas) and the average polices per capita in each city. The data includes six cities, including Taipei, Hsinchu, Taichung, Tainan, Hualien, and Kaohsiung, during the period of 1920–1941 (Fig. 1; Table 1).

**Table 1:** Descriptive statistics and correlations

Panel A. Descriptive statistics				
Variable	Mean	Std. Dev.	Min	Max
Log (land property lawsuits)	3.765	1.443	0	5.808
Log (rainfalls)	7.514	0.294	6.529	8.167
Log (population)	12.794	1.313	10.566	14.213
East region	0.328	0.471	0	1
Average police	0.004	0.003	0.001	0.013
Temperature	22.807	0.840	21.300	25
Evaporation	1491.742	273.427	716.500	2237.300

Panel B. Correlations				
Variable	Land property lawsuits	Rainfalls	Population	Average police
Land property lawsuits	1			
Rainfalls	0.105	1		
Population	0.253	−0.097	1	
Average police	−0.611	−0.087	−0.187	1

There are 128 samples, statistics in the top panel; bivariate correlations in the bottom panel

Figure 1 illustrates in detail the statistics represented by the functional form of logarithm, and the correlations between the rainfalls and the land property lawsuits. The pattern of rainfalls, which is showed on the top panel of Fig. 1, is more stable, and there exists a weakly positive correlation between the rainfalls and the land property lawsuits on the bottom panel of Fig. 1. This preliminary evidence indicates that rainfalls might affect the numbers of lawsuit and excluded the possible impacts from some extreme samples. In the panel B of Table 1, the variable of land property lawsuits is negatively associated to the average police. The results based on the correlations suggest that rainfalls may bring fewer impacts on land disputes. To further investigate the effects of rainfalls on the lawsuits, in the next section we applies ordinary least squares (OLS) and two stage least squares (2SLS) to assess whether rainfalls are significant.

## Empirical results

We set the empirical equation as follows:

1 $$\log \left( {{\text{land}}\,{\text{lawsuits}}_{\text{i}} } \right) = \gamma_{1} \,\log \left( {{\text{rainfalls}}_{\text{i}} } \right)\text{ + }\beta^{\prime}X_{i} \text{ + }\varepsilon_{i} ,$$

where X_i_ is the vector of explanatory variables. As mentioned by Brückner and Ciccone (2011). It pays more attention that the lawsuit variable in Taiwanese database is calculated by summarizing the recorded lawsuits in the different administrative areas. Because the statistical and historical restriction, it is impossible that the density or distribution of the data is described clearly. For example, city A maybe have a higher number of the lawsuits but these are distributed on some areas. At the same time, the data of lawsuits in city B are less than the city A but the density of data spreads uniformly over all areas in the city. Based on bindings of the historical data, a larger degree of volatility of the data will easily produce a biased estimator, which is called measurement error. Problems of measurement errors can be solved by adding instrument variables into the empirical regressions. According to the results from Murry (2006), the coefficients in estimated regressions are not significant if the instrument variables are added into these estimated regressions.

Following the concepts of Cornwell and Trumbull (1994) and Kelly (2000), suitable instrument variables should be exogenous, strongly related to the endogenous variables and uncorrelated to the error term of regressions. In this article, we will insert the variables of temperature and evaporation (unit: millimeter) as the instrument variables (Table 2).

**Table 2:** Estimated results

Dependent variable	Log (land property lawsuit)					
	OLS (1)	OLS (2)	OLS (3)	2SLS (1)	2SLS (2)	2SLS (3)
log (rainfalls)	−0.089	−0.320	−0.359	−1.088^b^	−1.707^b^	−1.885^a^
	(0.217)	(0.225)	(0.228)	(0.537)	(0.741)	(0.704)
log (population)	0.971^a^	1.244^a^	1.308^a^	1.029^a^	1.516^a^	1.667^a^
	(0.049)	(0.104)	(0.121)	(0.060)	(0.180)	(0.207)
East region		0.823^a^	0.498		1.432^a^	0.808
		(0.281)	(0.420)		(0.440)	(0.499)
Log (average police)			−0.284			−0.582
			(0.272)			(0.337)
Constant	−8.149	−10.016^a^	−8.736^a^	−1.232	−3.271	−0.172
	(1.618)	(1.690)	(2.087)	(3.822)	(3.883)	(4.380)
Sample	128	128	128	128	128	128
R-square	0.773	0.787	0.789	0.734	0.722	0.712
Score chi square				4.190	4.407	2.062
F statistics				8.757	6.192	7.112

Standard error in parentheses

^a^ Significant at 1 %

^b^ Significant at 5 %

Our main results are provided in Table 2. In Table 2, the dependent variable is the natural logarithm form of the land property lawsuits per year (that means the disputes on land property rights between landlords, tenants, trade conflicts and land easements etc.). In the first three column of Table 2, we investigate all the effects from explanatory variables by the main regressions. These results indicate that population variable is positive significantly related to the dependent variable, land lawsuits, and the coefficient of the rainfalls variable is negative but insignificant. These results probably represent a tradeoff between population and land resources, and then provide an incentive to define land rights or to provoke the disputes (Buoye, 2000).

The last three columns list the estimated results of 2SLS and indicate that our instrument variables are adequate. For example, in the second stage, score chi square on column 6 presents a coefficient of 2.062, which excluded instrument variables are correlated with the error term. More importantly, the F statistics is 7.112 and rejects the null hypothesis that assumes instrument variables are unrelated to the endogenous variables in the first stage estimation. Based on these test statistics, therefore, we conclude that our instrument variables are able to identify exogenous variation in the endogenous variables. It is comforting that the results under the second stage are consisting with OLS evidence, however, both population and rainfalls are matter but the influences on rainfalls are stronger and significant than OLS estimation.

The contribution of Brückner and Ciccone (2011) is that the rainfalls variable is a key factor which triggers democratic institutions in sub-Saharan regions with relatively scarce rainfalls. In other words, their results demonstrate that the lawsuit changes may be triggered by natural environment shocks. A negative shock from rainfalls will significantly reduce the land disputes if we can follow the arguments discussed by Brückner and Ciccone (2011), but, following our results, we find that a rainfall-abundant country will also decrease its social disputes or conflicts as long as rainfalls are more stable. However, in our considerations, we find that rainfalls have important roles in the process of economic development, especially in the economy which is based on agriculture as the mainstay such like Taiwan in the periods of Japanese colonial rule.

## Conclusions

In this article, we investigate the impacts of rainfalls on land lawsuits and discuss that how social stability in Taiwan changes with an exogenous natural shock. However, social stability is a measurement of indirect responses from changing in current economic environment, income, and growth. The variables using in this article are Taiwanese land-property rights, lawsuits in land trade and a statistic of rainfalls in 1920–1940. In addition, we choose the rainfalls as the climate variable when the lawsuit is chosen as a variable of social stability. Types of lawsuits in this article are included by the land, the item, and the money lawsuits. The reason that Taiwan is a research object is the period of switching from a pure agricultural economy to a mixed stage of agriculture-industry economy. At this time, it means that the market economy is emerging in Taiwan.

The influences about the land-property lawsuits in relations to rainfalls from the regression analysis: it shows no significant influences. An interesting thing is that, when we use temperature and evaporation as the instrument variables of rainfalls, we find that there exists a negative significant influence on quantities of land-property lawsuit. Which means that increase amount of rainfalls will show significant decreasing the quantity of land lawsuit cases. Therefore, the amount of rainfalls has direct or indirect influences on the value of lands. The empirical results from this article extend the ones from the past literature. Miguel et al. (2004), Ciccone (2011), Brückner and Ciccone (2011) studied the causal relationships of civic wars, economic shock, and democracy when the volatility of rainfalls becomes more and more unstable. In this article, we focus on an issue between rainfalls and property rights, and the evidences shows that a huge shock in values of lands closely depends on rainfalls in Taiwan from 1920 to 1940.

Here, we can make some conclusions. When a country is located on the agricultural or agriculture-industry stages of economic development, fluctuations form rainfalls will induce influences between economic outputs and values of lands. If 1 year has a low amount of rainfalls, it will decrease the available resources and increase the production costs, and then cause the disputes of land property rights. Our empirical results also show that the natural environment variables play an important role at the stage of modern economic beginning.

## References

[CR1] André C, Platteau JP (1998). Land relations under unbearable stress: Rwanda caught in the Malthusian trap. J Econ Behav Organ.

[CR2] Brückner M, Ciccone A (2011). Rain and the democratic window of opportunity. Econometrica.

[CR3] Buoye TM (2000). Manslaughter, markets, and moral economy: violent disputes over property rights in eighteenth-century China.

[CR4] Ciccone A (2011). Economic shocks and civil conflict: a comment. Am Econ J Appl Econ.

[CR5] Cornwell C, Trumbull WN (1994). Estimating the economic model of crime with panel data. Rev Econ Stat.

[CR6] Henderson VJ, Storeygard A, Weil DN (2012). Measuring economic growth from outer space. Am Econ Rev.

[CR7] Hodler R, Raschky PA (2014). Economic shocks and civil conflict at the regional level. Econ Lett.

[CR8] Kelly M (2000). Inequality and crime. Rev Econ Stat.

[CR9] Miguel E, Satyanath S, Sergenti E (2004). Economic shocks and civil conflict: an instrumental variables approach. J Polit Econ.

[CR10] Murry MP (2006). Avoiding invalid instruments and coping with weak instruments. J Econ Perspect.

[CR11] Xiao LB, Fang X, Zhang Y, Ye Y, Huang H (2014). Multi-stage evolution of social response to flood drought in the North China plain during 1644–1911. Reg Environ Chang.

